# Hepatitis delta virus-like circular RNAs from diverse metazoans encode conserved hammerhead ribozymes

**DOI:** 10.1093/ve/veab016

**Published:** 2021-02-18

**Authors:** Marcos de la Peña, Raquel Ceprián, John L Casey, Amelia Cervera

**Affiliations:** 1 IBMCP (CSIC-UPV), C/Ingeniero Fausto Elio s/n, Valencia 46022, Spain; 2 Department of Microbiology and Immunology, Georgetown University Medical Center, Washington, DC, USA

**Keywords:** circRNA, hepatitis delta virus, catalytic RNA

## Abstract

Human hepatitis delta virus (HDV) is a unique infectious agent whose genome is composed of a small circular RNA. Recent data, however, have reported the existence of highly divergent HDV-like circRNAs in the transcriptomes of diverse vertebrate and invertebrate species. The HDV-like genomes described in amniotes such as birds and reptiles encode self-cleaving RNA motifs or ribozymes similar to the ones present in the human HDV, whereas no catalytic RNA domains have been reported for the HDV-like genomes detected in metagenomic data from some amphibians, fish, and invertebrates. Herein, we describe the self-cleaving motifs of the HDV-like genomes reported in newts and fish, which belong to the characteristic class of HDV ribozymes. Surprisingly, HDV-like genomes from a toad and a termite show conserved type III hammerhead ribozymes, which belong to an unrelated class of catalytic RNAs characteristic of plant genomes and plant subviral circRNAs, such as some viral satellites and viroids. Sequence analyses revealed the presence of similar HDV-like hammerhead ribozymes encoded in two termite genomes, but also in the genomes of several dipteran species. *In vitro* transcriptions confirmed the cleaving activity for these motifs, with moderate rates of self-cleavage. These data indicate that all described HDV-like agents contain self-cleaving motifs from either the HDV or the hammerhead class. Autocatalytic ribozymes in HDV-like genomes could be regarded as interchangeable domains and may have arisen from cellular transcriptomes, although we still cannot rule out some other evolutionary explanations.

## 1. Introduction

The hepatitis delta or D virus (HDV) is a unique infectious agent whose genome is composed of a small (∼1,700 nt) single-stranded circRNA genome with high self-complementarity (Botelho-Souza et al. 2017). This satellite virus encodes only two proteins, the small and large hepatitis delta antigen (S- and L-HDAg), both derived from a single open reading frame. In addition, the HDV contains a characteristic self-cleaving RNA motif, the hepatitis delta virus ribozyme (HDVR), both in the genomic and antigenomic polarities (Kuo et al. 1988; Wu et al. 1989; Riccitelli and Lupták 2013). HDV depends on the hepatitis B virus (HBV) for assembly of viral particles, release from the host cell, and entry into new cells (Botelho-Souza et al. 2017).

Until recently, the HDV circRNA had only been detected in humans, and it is the sole representative of the *Deltavirus* genus. However, new data have revealed the presence of highly divergent HDV-like circRNAs in samples from diverse metazoan species, ranging from amniotes (reptiles, birds, and mammals) to amphibians (a newt and a toad) and invertebrates (a termite) (Wille et al. 2018; Chang et al. 2019; Hetzel et al. 2019; Paraskevopoulou et al. 2020; Bergner et al. 2021) indicating that this atypical virus has a longer and more complex evolutionary history than previously thought. Contrary to the human HDV, none of the newly described deltavirus-like genomes has been found associated with a coinfecting hepadnavirus. In that way, it has been proposed that HDV and metazoan HDV-like agents could use diverse helper viruses, such as arenavirus, vesiculovirus and other HBV-unrelated viruses, to acquire the envelope proteins necessary for transmission (Hetzel et al. 2019; Perez-Vargas et al. 2019). The newly described HDV-like agents share many characteristics with their human counterpart. They all have single-stranded circular RNA genomes of approximately 1,500-1,700 nt that fold into rod-like structures, and code for a putative delta antigen with 13–55 per cent amino acid identity to the human one. However, in the case of the HDVR motif, its presence has been reported for the HDV-like agents from amniotes (Wille et al. 2018; Hetzel et al. 2019; Paraskevopoulou et al. 2020), but not for those of amphibians, fish, and invertebrates (Chang et al. 2019). This fact is striking and worth further examination, since ribozymes are essential domains for processing and replication of the HDV circRNA through a rolling-circle mechanism (Flores et al. 2012; Harichandran et al. 2019).

**Figure 1.**
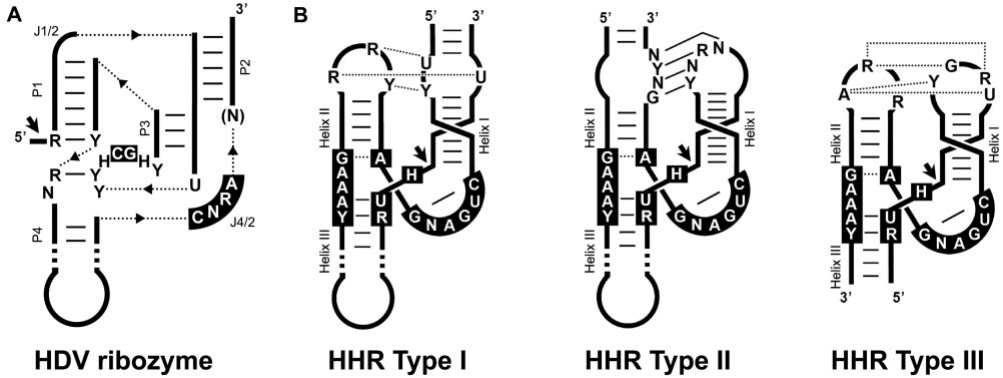
(A) Schematic representation of the general topology of the hepatitis delta virus ribozyme (HDVR). Dotted lines represent connections between the helixes. The most frequent conserved nucleotides are shown in black boxes. Helical stems (P) and single-stranded junction strands (J) are indicated. (B) Schematic representation of the three possible hammerhead ribozyme (HHR) topologies (Types I, II, and III). The most frequent nucleotides in the catalytic core are shown in black boxes. Conserved loop-loop interactions are indicated (dotted and continuous lines refer to non-canonical and Watson–Crick base pairs). Black arrows indicate the self-cleavage site. The three HHR types have been reported in prokaryotic/phage genomes, whereas metazoan and plant genomes mostly show type I and III motifs, respectively.

The ribozyme of the human hepatitis delta virus (HDV) or HDVR belongs to the family of small self-cleaving ribozymes, a group of RNA motifs that catalyse an intramolecular transesterification reaction in a sequence-specific manner (Ferré-D’Amaré and Scott 2010; Jimenez et al. 2015). Despite their very different structures, all nine classes of this family of ribozymes produce RNA fragments with 2′-3′-cyclic phosphate and 5′-hydroxyl ends after a nucleophilic attack by a 2′-oxygen group. In the case of the HDVR, both the genomic and antigenomic ribozymes show a characteristic nested double pseudoknot structure with five helical regions (Fig. 1A). A paradigmatic and well-studied member of the family of small self-cleaving RNAs is the hammerhead ribozyme (HHR) (Hutchins et al. 1986; Prody et al. 1986; de la Peña et al. 2017). The HHR is composed of three double helixes (I to III) that surround a core of 15 conserved nucleotides, and folds into a γ-shaped structure where the loops of helix I and II interact (de la Peña et al. 2003; Martick and Scott 2006). Depending on the open-ended helix, three circularly permuted topologies are possible for the HHR (type I, II, or III) (Fig. 1B).

Initially described in subviral agents with circRNA genomes—the HDV and some plant viral satellites and viroids—both HDVR and HHR motifs are now known to be pervasive in genomes throughout the tree of life (Webb et al. 2009; de la Peña and García-Robles 2010). In many instances, these ribozymes are associated with retrotransposable elements, and are thought to play essential roles in their life cycle. Diverse non-LTR transposons, such as R2, RTEs, and other LINEs, possess active HDVR motifs encoded in their 5′ termini (Eickbush and Eickbush 2010; Ruminski et al. 2011; Sánchez-Luque et al. 2011). HHR motifs, on the other hand, have been found embedded within the terminal repeats of *Penelope*-like elements (Cervera and de la Peña 2014), *Terminon* giant retrotransposons (Arkhipova et al. 2017), and non-autonomous LTR and non-LTR retrozymes of plants and animals, respectively (Cervera et al. 2016; Cervera and de la Peña 2020). Interestingly, the retrotransposition intermediates of both LTR and non-LTR *retrozymes* are small circular RNAs that accumulate at high levels in different organisms, and share many characteristics with infectious HHR-containing circRNAs of plants (de la Peña and Cervera 2017). The conserved presence of diverse self-cleaving ribozymes in infectious agents as well as in retrotransposons hints at a complex evolutionary relationship among all these mobile elements (de la Peña et al. 2020).

In this paper, we analyse the metagenomic HDV-like sequences of newts, toads, fish, and termites reported by Chang et al. (2019) for the presence of small self-cleaving ribozymes.

## 2. Materials and Methods

### 2.1 Bioinformatics and sequence analysis

Sequence homology searches through BLAT (Kent 2002), BLASTX, and BLAST (Altschul et al. 1990) tools were carried out in individual or grouped genomes, and in specific BioSample accessions (SAMN11445145 and SAMN11445146). Sequence alignments were performed with ClustalX (Larkin et al. 2007), MUSCLE (Edgar 2004), and Jalview (Waterhouse et al. 2009) software. The RNAmotif (Macke et al. 2001) and InfeRNAl (Nawrocki and Eddy 2013) software was used for the detection of HDVR and HHR motifs with an extended type III architecture. Secondary RNA structures of minimum free energy were calculated with the RNAfold program from the ViennaRNA Package (Lorenz et al. 2011).

### 2.2 Sequence cloning and transcription

**Figure 2.**
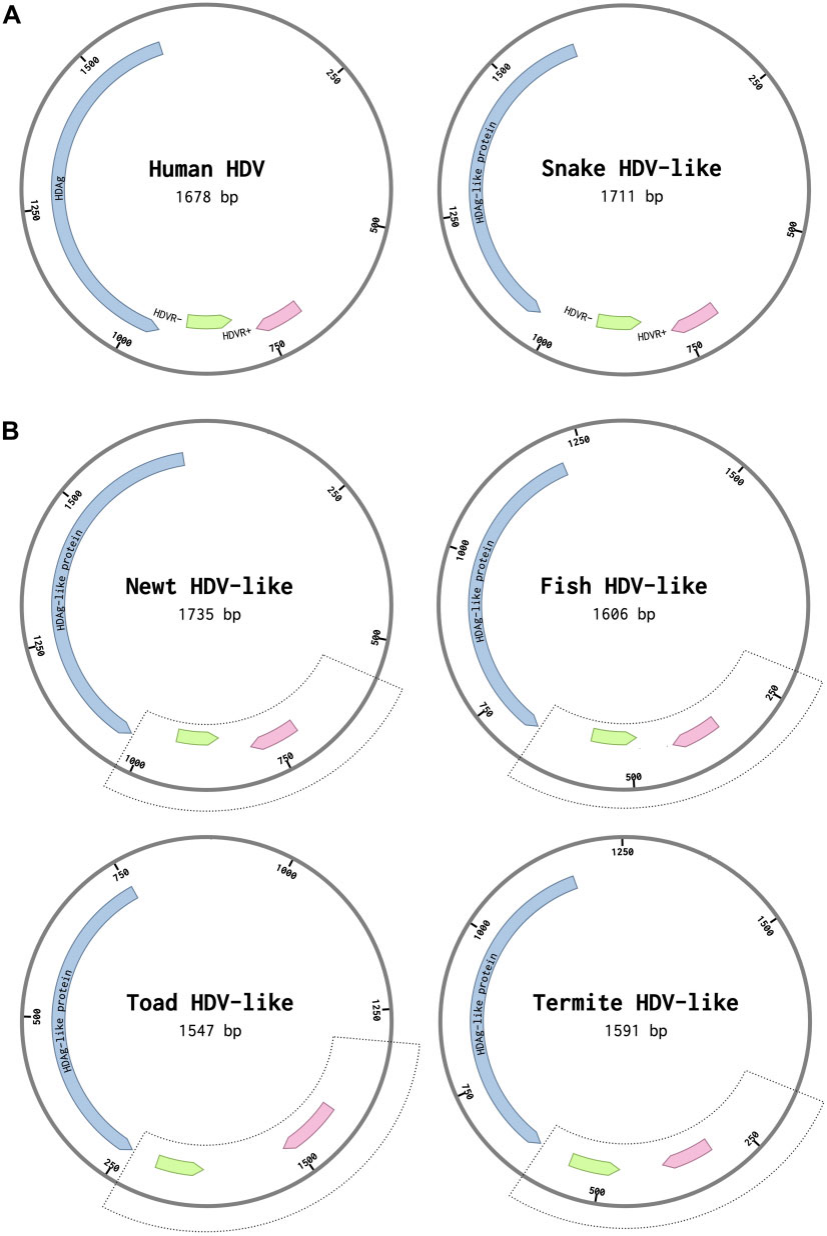
(A) Genome organization of the human HDV and the *boa constrictor* snake HDV-like circRNAs. Delta antigen-coding, genomic, and antigenomic HDV ribozyme regions (in blue, pink, and green, respectively) are indicated. (B) Newt, fish, toad, and termite HDV-like circRNAs. The regions used for sequence analysis are showed in dotted line boxes. The putative regions corresponding to genomic and antigenomic self-cleaving motifs are indicated (GenBank accession numbers: human X04451.1, snake MH988742.1, newt MN031239.1, fish MN031240.1, toad MK962760.1, termite MK962759.1).

Sequences corresponding to the newt and fish HDV-like regions of interest (Fig. 2) and to the toad and termite HDV-like antigenomic HHRs preceded by the T7 RNA polymerase promoter (Fig. 6) were purchased as gBlock Gene Fragments (Integrated DNA Technologies). DNA fragments were cloned into a properly linearized pBlueScript KS or pUC18 vectors by cohesive-end ligations. RNAs of the cloned sequences were synthesized by *in vitro* run-off transcription of the linearized plasmids containing either the HDV-like fragment or the HHR motifs. The transcription reactions contained 40 mM Tris–HCl, pH 8, 6 mM MgCl_2_, 2 mM spermidine, 0.5 mg/ml RNase-free bovine serum albumin, 0.1% Triton X-100, 10 mM dithiothreitol, 1 mM each of ATP, CTP, GTP, and UTP, 2 U/µl of Ribonuclease Inhibitor (Takara Inc), 20 ng/µl of plasmid DNA, and 4 U/µl of T7 or T3 RNA polymerases. After incubation at 37°C during the indicated time, the products were fractionated by polyacrylamide gel electrophoresis (PAGE) in 5 per cent (HDV-like fragments) or 15 per cent gels (HHRs) with 8 M urea.

### 2.3 Self-cleavage analyses

Analyses of HHR self-cleavage activity under co-transcriptional conditions were performed as previously described (Long and Uhlenbeck 1994). Appropriate aliquots of the transcription reactions (smaller volumes were taken at longer incubation times) were removed at different time intervals, quenched with a fivefold excess of stop solution at 0°C, and analysed as previously described (Long and Uhlenbeck 1994). Briefly, the uncleaved and cleaved transcripts were separated by PAGE in 15 per cent denaturing gels and detected by Sybr Gold staining (Thermo Fisher Scientific). The product fraction at different times, F_t_, was determined by quantitative scanning of the corresponding gel bands and fitted to the equation F_t_ = F_∞_(1 − e^–kt^), where F_∞_ is the product fraction at the reaction endpoint, and k is the first-order rate constant of cleavage (k_obs_).

## 3. Results

### 3.1 Bioinformatic detection of self-cleaving motifs in HDV-like circRNAs

It has been previously reported the existence of divergent HDV-like agents in metagenomic data obtained from disparate metazoans such as a newt (the Chinese fire belly newt *Cynops orientalis*), a toad (the Asiatic toad *Bufo gargarizans*), fish (a mixture from classes *Actinopterygii, Chondrichthyes*, and *Agnatha*), and a termite (*Schedorhinotermes intermedius*) species (Chang et al. 2019). The sequences obtained corresponded to circRNAs with similar sizes to the human HDV virus (∼1,500–1,700 nt) and are also predicted to adopt classical rod-like secondary structures. Moreover, the four metazoan HDV-like genomes encode highly divergent copies of the delta antigen ORF. However, the presence of the HDV self-cleaving domains or HDVRs were not reported for any of these genomes, and it has been suggested that these novel HDV-like circRNAs may lack the ribozyme motifs (Paraskevopoulou et al. 2020).

**Figure 3.**
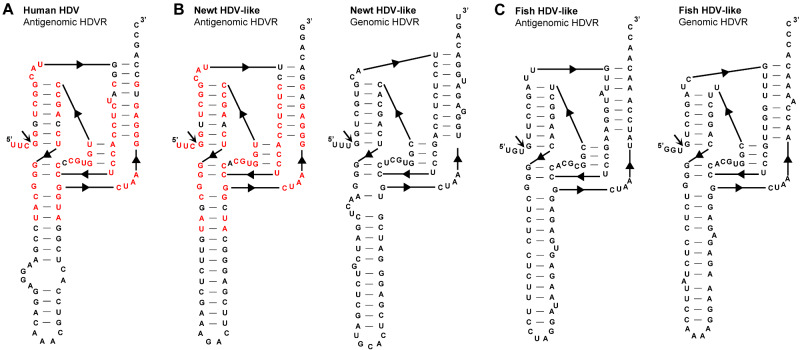
(A) Secondary structure of an antigenomic human hepatitis delta virus ribozyme (HDVR) sequence (GenBank accession number MG711754.1). The secondary structure of the antigenomic (left) and genomic (right) HDVRs present in HDV-like genomes of (B) newt and (C) a mix of fish. Sequence identity between the antigenomic HDVRs from human HDV and the newt HDV-like agent are highlighted in red nucleotides.

To confirm the presence or absence of self-cleaving motifs in these HDV-like genomes, homology-based searches were initially performed. Based on the typical location of genomic and antigenomic ribozymes present in other HDV agents from humans and amniotes (Fig. 2A), we focused on the regions (∼400 bp) immediately following the predicted delta antigen coding sequences (Fig. 2B). BLAST-searches showed that a portion at the 5′ region of the newt HDV-like sequence clearly mapped to the 5′ end of the antigenomic Human HDVR motif, strongly suggesting the presence of HDVR-like motifs in the newt sequence (Supplementary Fig. S1A). Sequence alignments followed by manual inspection of the regions of interest from the newt HDV-like sequence confirmed the presence of HDVRs in both the genomic and antigenomic strands (Supplementary Fig. S1B and Fig. 3B). Similar sequence analysis revealed the presence of analogous HDVR motifs in the fish HDV-like circRNA (Supplementary Fig. S1C and Fig. 3C). Apart from the antigenomic newt and human HDVR motifs (Fig. 3), the rest of the detected ribozymes did not show sequence homology with other sequences in the GeneBank database. However, newt and fish HDVRs show similar architecture and helix sizes to those described for the human HDVR motifs (Figs 1A and 3): five paired regions forming two coaxial stacks, P1(∼7 bp)/P4(∼12 bp) and P2(∼8 bp)/P3(3 bp), linked by short single-stranded joining strands J1/2(2 nt) and J4/2(4–6 nt).

**Figure 4.**
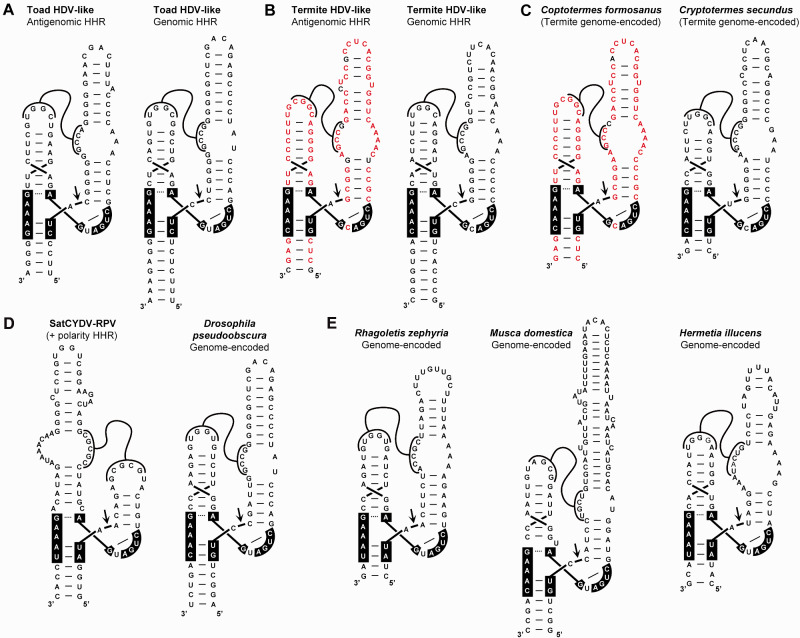
Secondary structure of the antigenomic and genomic hammerhead ribozymes (HHRs) present in the HDV-like genomes of (A) a toad and (B) a termite. (C) Examples of similar HHRs encoded in the DNA genomes of the termite *Coptotermes formosanus* (BLKM01010448.1) and *Cryptotermes secundus* (NEVH01024570.1) species. Sequence identity between the antigenomic HHR from the termite HDV-like RNA and a DNA genome-encoded HHR motif in *C. formosanus* termite are highlighted in red. (D) Secondary structure of the HHR motifs present in the circRNA Satellite (+ polarity) of the Cereal Yellow Dwarf Virus serotype RPV (SatCYDV-RPV, GenBank: M63666.1) and in the *D. pseudoobscura* genome (Perreault et al. 2011). (E) Examples of HHRs motifs detected in the genomes of the fruit fly *Rhagoletis zephyria*, the housefly *Musca domestica* and the soldier fly *Hermetia illucens* (de la Peña et al., in preparation).

Analysis of the 400 nt regions from the toad and termite HDV-like circRNAs did not reveal any HDVR motif. However, alignments of the genomic and antigenomic regions from toad and termite surprisingly showed the presence of the short conserved sequences compatible with the well-known HHR motif (Supplementary Fig. S1D and Fig. 1B). The secondary structure of the genomic and antigenomic toad and termite HHRs have a very similar architecture, which corresponds to an atypical class of type III HHR characterized by larger than usual helixes I and II (Fig. 4A and B). Most type III HHRs, as those present in plant retrozymes and in subviral agents, have a helix I with a 6 base paired stem capped by a 6–8 nt loop, which interacts with a 4 nt loop of helix II (typically with a 4-bp stem) through a set of conserved non-Watson–Crick tertiary interactions (Fig. 1B) (de la Peña et al. 2017). However, the atypical type III HHRs detected in toad and termite HDV-like circRNAs show a much longer helix I with a first shorter stem (5 bp instead of 6 bp), followed by an internal loop (6–10 nt), and capped by an extra RNA hairpin of about 20 extra nt. Interestingly, the internal loop in all four hammerheads show a conserved 5′ sequence (consensus sequence GCCR, where R stands for a purine), which can interact through Watson–Crick/wobble base pairs with the loop of helix II (a 6-bp stem capped by a loop carrying the consensus sequence YGGC). At least two examples of similar type III HHRs with longer helixes and stable pseudoknots between loops 1 and 2 have been previously described in the literature (Fig. 4D). Interestingly, one of these motifs corresponds to a genomic HHR detected in *Drosophila pseudoobscura* (Perreault et al. 2011), which shows a highly similar architecture to the toad and termite HDV-like HHRs, despite a low sequence homology among them.

Another feature observed in these HDV-like HHR sequences is the atypical cleavage sites of both genomic and antigenomic ribozyme motifs of the toad HDV-like agent. Most HHRs described in the literature have a cleavage site with the consensus sequence RUH (where R stands for a purine and H for any nucleotide excepting G), whereas the toad HDV-like HHRs show the unusual ‘cUC’ and ‘cUA’ cleavage sites, which are, in any case, properly closed with a perfect stem III (‘GAAAg’ box in the toad HDV-like HHRs, instead the consensus ‘GAAAY’ box sequence conserved in most HHRs. Fig. 1B) (de la Peña et al. 2017).

### 3.2 Phylogenetically related HDV-like HHRs encoded in the DNA genomes of diverse termite and dipteran species

The sequences of the HHR motifs detected in the toad and termite HDV-like genomes were used as seed queries for homology-based searches in genomic databases. No clear hits were obtained using any of the toad HHR motifs. However, BLAST searches with the antigenomic HHR of the termite HDV-like genome revealed the presence of two highly similar HHR sequences in the genome of the termite *Coptotermes formosanus* (Fig. 4C, left). Similarly, BLAST searches with the genomic HDV-like HHR of the termite identified another two HHR motifs in the genome of the termite *Cryptotermes secundus* (Fig. 4C, right). These genomic HHRs from both *Coptotermes* and *Cryptotermes spp.* were found as isolated motifs within different large contigs (∼20 kb to 2 Mb). In the surrounding 5′- and 3′-regions (2 kb) of the genomic HHRs of termites we did not detect any sequences homologous to HDV-like sequences nor even any recognizable ORFs, but rather only similarities to genomic sequences from other termite species. Structure-based searches based on this atypical type III architecture did not reveal additional examples of similar HHRs in termite genomes, nor in some other related insects such as hymenopterans, hemipterans, and a cockroach. However, more extensive bioinformatic searches among distant invertebrates unveiled the occurrence of different examples of type III HHRs structurally similar to the ones present in HDV-like agents and *D. pseudoobscura* genome. These new motifs were mostly found in the genomes of dipterans, such as the common housefly or diverse fruit fly spp., among others (Fig. 4E. de la Peña et al., in preparation).

### 3.3 Cloning and self-cleavage analysis

**Figure 5.**
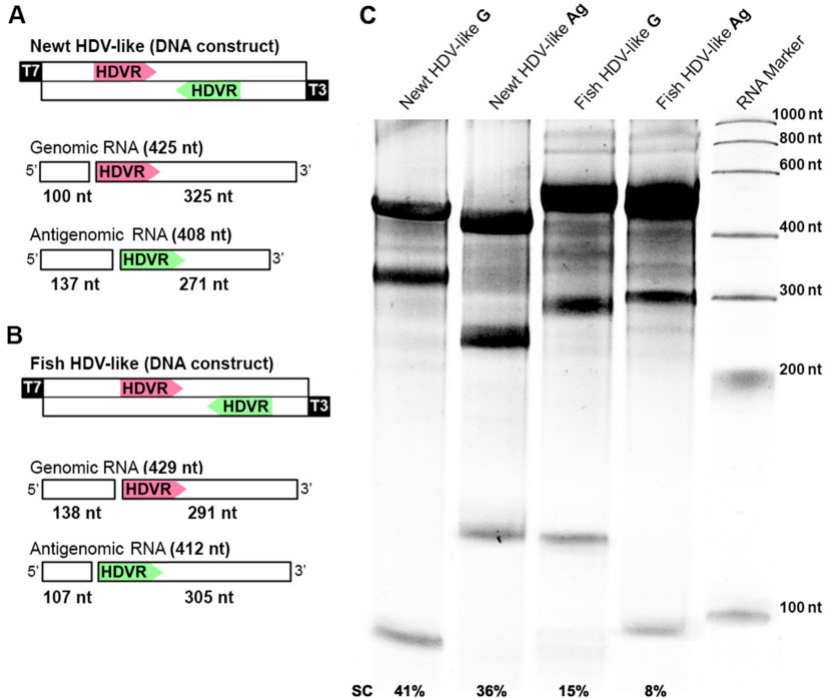
(A) Schematic representation of the DNA construct (top) and the resulting genomic and antigenomic transcripts (bottom) corresponding to the newt HDV-like region of interest (see Fig. 2). The size of the primary transcripts and the two expected products of ribozyme self-cleavage are indicated. (B) The same scheme as before for the case of the fish HDV-like sequence. (C) Denaturing PAGE of the resulting RNA products from run-off transcriptions (Both genomic/G and antigenomic/Ag polarities) of the constructs from newt and fish HDV-like sequences. The sizes of the primary bands and cleavage products are shown in panels (A) and (B). The percentage of self-cleavage (SC) is shown at the bottom.

Regions of about 400 bp corresponding to the newt and fish HDV-like genomes harboring both genomic and antigenomic HDV ribozymes (Fig. 2B) were cloned under the control of T7/T3 transcriptional promoters (Fig. 5A and B). RNA products obtained after run-off transcriptions (1 h) of each polarity and animal species showed the fragments with the expected sizes due to the ribozymes cleavage (Fig. 5C). Both newt HDVRs reached a final ∼40 per cent self-cleavage of the primary transcript, whereas fish HDRVs showed lower levels of cleavage (15 and 8 per cent cleavage of the primary genomic and antigenomic transcript sequences, respectively). The observed self-cleavage products confirm the presence of the HDVRs in both polarities of newt and fish HDV-like circRNAs, whereas the differences in the cleavage ratios may indicate distinct ribozyme efficiencies, but also differences in the effect of 3′ competing sequences (see below and Supplementary Fig. S2) (Diegelman-Parente and Bevilacqua 2002).

**Figure 6.**
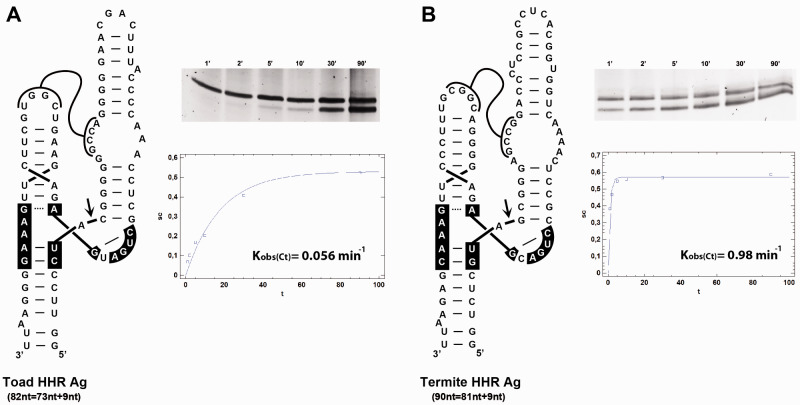
Self-cleavage kinetic analysis of the antigenomic HHR ribozymes detected in (A) toad and (B) termite HDV-like sequences. A representative example of a 15% PAGE with a kinetic analysis and quantification graph of each HHR co-transcriptional cleavage is shown on the right.

Minimal constructs carrying the antigenomic HHR sequence of toad and termite HDV-like genomes were cloned under the control of the T7 RNA promoter. As shown in Figure 6, both HHR motifs show clear self-cleaving activity during transcription. However, the analysis of co-transcriptional cleavage of the HHR motif from the toad HDV-like RNA indicates a much lower self-cleavage efficiency (k_obs_=0.05 ± 0.01 min^−1^) compared with the values obtained for the termite HHR (k_obs_=0.96 ± 0.09 min^−1^). This difference in the catalytic efficiency of toad and termite HHRs (20-fold) is in agreement with the observed sequence differences in the site of self-cleavage of the toad motif (see above), which is known to deeply reduce the self-cleaving capability of the HHR (Ruffner et al. 1990). Moreover, in both cases we observed that HHR cleavage reached lower completion values (50–60%) compared with the levels observed for typical type III HHRs (above 90%) (de la Peña et al. 2003).

## 4. Discussion

In contrast with the ubiquitous circular DNAs such as most prokaryotic, plastid, and bacteriophage genomes, covalently closed circular RNAs had been regarded as exceptional nucleic acids until very recently. We know now the existence of many types of circRNAs, from the splicing-derived circRNAs (Li et al. 2018) to the eukaryotic retrozymes (Cervera et al. 2016; Cervera and de la Peña 2020), or the heterogenous family of infectious circRNA genomes (Flores et al. 2016; de la Peña et al. 2020). Diverse members of this latter group of infectious agents are characterized by the presence of autocatalytic ribozymes, which are required for the replication of their circular RNA genomes through a rolling-circle mechanism. In plant viral satellites and some viroids, a common ribozyme is the HHR, although three viral satellites also show the presence of a rare class of small catalytic RNA, the hairpin ribozyme (Fedor 2000). On the other hand, human HDV and novel HDV-like agents from amniotes, fish, and newts contain a third class of small ribozyme, the HDVR. All these small ribozyme motifs do not seem to show any clear sequence or structural relationship among them, and for that reason, the presence of conserved type III HHRs in the HDV-like agents of a toad and a termite is surprising, and somehow connects a bit more the infectious circRNAs of plants (viroidal agents) and animals (Deltavirus agents). However, although HHRs in HDV-like circRNAs can be regarded as analogous motifs to type III HHRs of plant subviral agents and retrozymes, their different topologies and tertiary interactions do not allow us to confirm a direct evolutionary relationship among them. Alternatively, these exceptional HDV-like HHRs may represent a case of convergence and, as proposed for some subviral circRNA agents of plants, the origin of these self-cleaving motifs could be in the cell transcriptomes (de la Peña and Cervera 2017). Previous data have confirmed that both HDVR and HHR are ubiquitous ribozymes encoded in DNA genomes from prokaryotes to humans. These motifs are commonly involved in diverse families of mobile genetic retroelements, which in some cases are expressed as abundant circRNAs. We can envisage that this population of ribozyme-containing RNAs could be a suitable source for the appearance of novel infectious agents of circRNA, either as viral satellites (through virus encapsidation of the ribozyme-containing RNA during cell infection) or as autonomous agents. In this line, other widespread self-cleaving ribozymes, such as the twister ribozyme (Roth et al. 2014) among others, may also occur in new HDV-like or any similar infectious circRNAs to be discovered. The presence of phylogenetically related HHRs in both HDV-like agents and invertebrate genomes (such as termites and dipterans) could support these hypotheses. Nevertheless, and in the absence of any clear evolutionary origin for the small ribozymes present in eukaryotes, we cannot rule out the opposite possibility where infectious circRNAs from unknown natural reservoirs would be the source of the self-cleaving ribozymes widespread among eukaryotic genomes.

Our data indicate that, from an evolutionary point of view, both HHR and HDVR can provide the cleavage function essential for double rolling circle replication of HDV-like circRNAs and could be regarded as interchangeable motifs among members of this family. Previous data reported that human HDV can be circularized in host cells after being processed by any self-cleaving motif capable of producing 5′-OH and 2′-3′-cyclic phosphate ends, either HDVR or HHR (Reid and Lazinski 2000). Moreover, our co-transcriptional self-cleaving analyses suggest that the intrinsic catalytic activity of the HHR ribozymes would not be crucial for the agent viability, and that a wide range of values can be tolerated depending on the HDV-like genome. Co-transcriptional self-cleavage analysis of the human HDVR performed under similar conditions resulted in a k_obs_∼0.4 min^−1^ (Diegelman-Parente and Bevilacqua 2002), which is in the same order than the termite HDV-like HHR and one order of magnitude faster than the toad HDV-like HHR. It has to be pointed out, however, that both HDVRs and HHRs occur in very similar genomic regions of the circRNAs (Fig. 2). This conserved arrangement together with the sequence homology between genomic and antigenomic ribozymes for each HDV-like genome, indicates that self-cleavage activity could be somehow regulated by the 3′-competing structures, as previously proposed for the human HDV (Diegelman-Parente and Bevilacqua 2002) (Supplementary Fig. S2).

The newly discovered HDV-like circRNAs in vertebrate and invertebrate species are also opening an intriguing door regarding whether these novel elements are either new virus satellites or viroid-like agents, and their possible origins. Future research will allow us to understand the biology and evolutionary relationships of this fascinating family of minimal infectious agents with circular RNA genomes.

## Data availability

All the sequences analyzed in this study were downloaded from the NCBI GenBank.

## Funding

Funding for this work was provided by the Ministerio de Economía y Competitividad of Spain and FEDER funds (BFU2017-87370-P) to M.d.l.P., and by NIH NIAID R21 AI149049 grant to J.L.C.

## Supplementary Material

veab016_Supplementary_Data

## References

[veab016-B1] Altschul S. F. et al (1990) ‘ Basic Local Alignment Search Tool’, Journal of Molecular Biology, 215: 403–10. 2231712 10.1016/S0022-2836(05)80360-2

[veab016-B2] Arkhipova I. R. , Yushenova I. A., Rodriguez F. (2017) ‘ Giant Reverse Transcriptase-Encoding Transposable Elements at Telomeres’, Molecular Biology and Evolution, 34: 2245–57. 28575409 10.1093/molbev/msx159 PMC5850863

[veab016-B3] Bergner L. et al (2021) ‘ Diversification of Mammalian Deltaviruses by Host Shifting’, Proceedings of the National Academy of Sciences of the United States of America, 118: e2019907118. 33397804 10.1073/pnas.2019907118 PMC7826387

[veab016-B4] Botelho-Souza L. F. et al (2017) ‘ Hepatitis Delta: Virological and Clinical Aspects’, Virology Journal, 14: 1–15. 28903779 10.1186/s12985-017-0845-y PMC5597996

[veab016-B5] Cervera A. , de la Peña M. (2014) ‘ Eukaryotic Penelope-like Retroelements Encode Hammerhead Ribozyme Motifs’, Molecular Biology and Evolution, 31: 2941–7. 25135949 10.1093/molbev/msu232 PMC4209133

[veab016-B6] Cervera A. , de la Peña M. (2020) ‘ Small circRNAs with Self-Cleaving Ribozymes Are Highly Expressed in Diverse Metazoan Transcriptomes’, Nucleic Acids Research, 48: 5054–64. 32198887 10.1093/nar/gkaa187 PMC7229834

[veab016-B7] Cervera A. , Urbina D., de la Peña M. (2016) ‘ Retrozymes Are a Unique Family of Non-Autonomous Retrotransposons with Hammerhead Ribozymes That Propagate in Plants through Circular RNAs’, Genome Biology, 17: 135. 27339130 10.1186/s13059-016-1002-4 PMC4918200

[veab016-B8] Chang W.-S. et al (2019) ‘ Novel Hepatitis D-like Agents in Vertebrates and Invertebrates’, Virus Evolution, 5: vez021. 31321078 10.1093/ve/vez021 PMC6628682

[veab016-B9] de la Peña M. , Ceprián R., Cervera A. (2020) ‘ A Singular and Widespread Group of Mobile Genetic Elements: RNA Circles with Autocatalytic Ribozymes’, Cells, 9: 2555. 10.3390/cells9122555 PMC7761336 33260527

[veab016-B10] de la Peña M. , Cervera A. (2017) ‘ Circular RNAs with Hammerhead Ribozymes Encoded in Eukaryotic Genomes: The Enemy at Home’, RNA Biology, 14: 985–91. 28448743 10.1080/15476286.2017.1321730 PMC5680766

[veab016-B11] de la Peña M. , Gago S., Flores R. (2003) ‘ Peripheral Regions of Natural Hammerhead Ribozymes Greatly Increase Their Self-Cleavage Activity’, The EMBO Journal, 22: 5561–70. 14532128 10.1093/emboj/cdg530 PMC213784

[veab016-B12] de la Peña M. , García-Robles I., Cervera A. (2017) ‘ The Hammerhead Ribozyme: A Long History for a Short RNA’, Molecules, 22: 78–11. 10.3390/molecules22010078 PMC6155905 28054987

[veab016-B13] de la Peña M. , García-Robles I. (2010) ‘ Ubiquitous Presence of the Hammerhead Ribozyme Motif along the Tree of Life’, RNA (New York, N.Y.), 16: 1943–50. 10.1261/rna.2130310 PMC2941103 20705646

[veab016-B14] Diegelman-Parente A. , Bevilacqua P. C. (2002) ‘ A Mechanistic Framework for Co-Transcriptional Folding of the HDV Genomic Ribozyme in the Presence of Downstream Sequence’, Journal of Molecular Biology, 324: 1–16. 12421555 10.1016/s0022-2836(02)01027-6

[veab016-B15] Edgar R. C. (2004) ‘ MUSCLE: Multiple Sequence Alignment with High Accuracy and High Throughput’, Nucleic Acids Research, 32: 1792–7. 15034147 10.1093/nar/gkh340 PMC390337

[veab016-B16] Eickbush D. G. , Eickbush T. H. (2010) ‘ R2 Retrotransposons Encode a Self-Cleaving Ribozyme for Processing from an rRNA Cotranscript’, Molecular and Cellular Biology, 30: 3142–50. 20421411 10.1128/MCB.00300-10 PMC2897577

[veab016-B17] Fedor M. J. (2000) ‘ Structure and Function of the Hairpin Ribozyme’, Journal of Molecular Biology, 297: 269–91. 10715200 10.1006/jmbi.2000.3560

[veab016-B18] Ferré-D’Amaré A. R. R. , Scott W. G. G. (2010) ‘ Small Self-Cleaving Ribozymes’, Cold Spring Harbor Perspectives in Biology, 2: a003574–a003574. 20843979 10.1101/cshperspect.a003574 PMC2944367

[veab016-B19] Flores R. , Ruiz-Ruiz S., Serra P. (2012) ‘ Viroids and Hepatitis Delta Virus’, Seminars in Liver Disease, 32: 201–10. 22932968 10.1055/s-0032-1323624

[veab016-B20] Flores R. , Owens R. A., Taylor J. (2016) ‘ Pathogenesis by Subviral Agents: Viroids and Hepatitis Delta Virus’, Current Opinion in Virology, 17: 87–94. 26897654 10.1016/j.coviro.2016.01.022

[veab016-B21] Harichandran K. et al (2019) ‘ Hepatitis Delta Antigen Regulates mRNA and Antigenome RNA Levels during Hepatitis Delta Virus Replication’, Journal of Virology, 93: e01989-18. 30728256 10.1128/JVI.01989-18 PMC6450126

[veab016-B22] Hetzel U. et al (2019) ‘ Identification of a Novel Deltavirus in Boa Constrictors’, MBio, 10: 1–8. 10.1128/mBio.00014-19 PMC6445931 30940697

[veab016-B23] Hutchins C. J. et al (1986) ‘ Self-Cleavage of plus and minus RNA Transcripts of Avocado Sunblotch Viroid’, Nucleic Acids Research, 14: 3627–40. 3714492 10.1093/nar/14.9.3627 PMC339804

[veab016-B24] Jimenez R. M. , Polanco J. A., Lupták A. (2015) ‘ Chemistry and Biology of Self-Cleaving Ribozymes’, Trends in Biochemical Sciences, 40: 648–61. 26481500 10.1016/j.tibs.2015.09.001 PMC4630146

[veab016-B25] Kent W. J. (2002) ‘ BLAT–the BLAST-like Alignment Tool’, Genome Research, 12: 656–64. 11932250 10.1101/gr.229202 PMC187518

[veab016-B26] Kuo M. Y. et al (1988) ‘ Characterization Self-Cleaving Antigenome Hepatitis’, Journal of Virology, 62: 4439–44. 3184270 10.1128/jvi.62.12.4439-4444.1988 PMC253552

[veab016-B27] Larkin M. A. et al (2007) ‘ Clustal W and Clustal X Version 2.0’, Bioinformatics, 23: 2947–8. 17846036 10.1093/bioinformatics/btm404

[veab016-B28] Li X. , Yang L., Chen L. L. (2018) ‘ The Biogenesis, Functions, and Challenges of Circular RNAs’, Molecular Cell, 71: 428–42. 30057200 10.1016/j.molcel.2018.06.034

[veab016-B29] Long D. M. , Uhlenbeck O. C. (1994) ‘ Kinetic Characterization of Intramolecular and Intermolecular Hammerhead RNAs with Stem II Deletions’, Proceedings of the National Academy of Sciences of the United States of America, 91: 6977–81. 7518924 10.1073/pnas.91.15.6977 PMC44321

[veab016-B30] Lorenz R. et al (2011) ‘ ViennaRNA Package 2.0’, Algorithms for Molecular Biology, 6: 26. 22115189 10.1186/1748-7188-6-26 PMC3319429

[veab016-B31] Macke T. J. et al (2001) ‘ RNAMotif, an RNA Secondary Structure Definition and Search Algorithm’, Nucleic Acids Research, 29: 4724–35. 11713323 10.1093/nar/29.22.4724 PMC92549

[veab016-B32] Martick M. , Scott W. G. (2006) ‘ Tertiary Contacts Distant from the Active Site Prime a Ribozyme for Catalysis’, Cell, 126: 309–20. 16859740 10.1016/j.cell.2006.06.036 PMC4447102

[veab016-B33] Nawrocki E. P. , Eddy S. R. (2013) ‘ Infernal 1.1: 100-Fold Faster RNA Homology Searches’, Bioinformatics, 29: 2933–5. 24008419 10.1093/bioinformatics/btt509 PMC3810854

[veab016-B34] Paraskevopoulou S. et al (2020) ‘ Mammalian Deltavirus without Hepadnavirus Coinfection in the Neotropical Rodent Proechimys Semispinosus’, Proceedings of the National Academy of Sciences of the United States of America, 117: 17977–83. 32651267 10.1073/pnas.2006750117 PMC7395443

[veab016-B35] Perez-Vargas J. et al (2019) ‘ Enveloped Viruses Distinct from HBV Induce Dissemination of Hepatitis D Virus in Vivo’, Nature Communications, 10: 2098. 10.1038/s41467-019-10117-z PMC6506506 31068585

[veab016-B36] Perreault J. et al (2011) ‘ Identification of Hammerhead Ribozymes in All Domains of Life Reveals Novel Structural Variations’, PLoS Computational Biology, 7: e1002031. 21573207 10.1371/journal.pcbi.1002031 PMC3088659

[veab016-B37] Prody G. A. et al (1986) ‘ Autolytic Processing of Dimeric Plant Virus Satellite RNA’, Science, 231: 1577–80. 17833317 10.1126/science.231.4745.1577

[veab016-B38] Reid C. E. , Lazinski D. W. (2000) ‘ A Host-Specific Function is Required for Ligation of a Wide Variety of Ribozyme-Processed RNAs’, Proceedings of the National Academy of Sciences of the United States of America, 97: 424–9. 10618434 10.1073/pnas.97.1.424 PMC26679

[veab016-B39] Riccitelli N. , Lupták A. (2013) ‘ HDV Family of Self-Cleaving Ribozymes’, Progress in Molecular Biology and Translational Science, 120: 123–71. 24156943 10.1016/B978-0-12-381286-5.00004-4

[veab016-B40] Roth A. et al (2014) ‘ A Widespread Self-Cleaving Ribozyme Class is Revealed by Bioinformatics’, Nature Chemical Biology, 10: 56–60. 24240507 10.1038/nchembio.1386 PMC3867598

[veab016-B41] Ruffner D. E. , Uhlenbeck O. C., Stormo G. D. (1990) ‘ Sequence Requirements of the Hammerhead RNA Self-Cleavage Reaction’, Biochemistry, 29: 10695–702. 1703005 10.1021/bi00499a018

[veab016-B42] Ruminski D. J. et al (2011) ‘ Processing and Translation Initiation of Non-Long Terminal Repeat Retrotransposons by Hepatitis Delta Virus (HDV)-like Self-Cleaving Ribozymes’, Journal of Biological Chemistry, 286: 41286–95. 10.1074/jbc.M111.297283 PMC3308841 21994949

[veab016-B43] Sánchez-Luque F. J. et al (2011) ‘ Identification of an Hepatitis Delta Virus-like Ribozyme at the mRNA 5′-End of the L1Tc Retrotransposon from Trypanosoma cruzi’, Nucleic Acids Research, 39: 8065–77. 21724615 10.1093/nar/gkr478 PMC3185411

[veab016-B44] Waterhouse A. M. et al (2009) ‘ Jalview Version 2-a Multiple Sequence Alignment Editor and Analysis Workbench’, Bioinformatics, 25: 1189–91. 19151095 10.1093/bioinformatics/btp033 PMC2672624

[veab016-B45] Webb C. H. T. et al (2009) ‘ Widespread Occurrence of Self-Cleaving Ribozymes’, Science, 326: 953. 19965505 10.1126/science.1178084 PMC3159031

[veab016-B46] Wille M. et al (2018) ‘ A Divergent Hepatitis D-like Agent in Birds’, Viruses, 10: 720. 10.3390/v10120720 PMC6315422 30562970

[veab016-B47] Wu H. N. et al (1989) ‘ Human Hepatitis δ Virus RNA Subfragments Contain an Autocleavage Activity’, Proceedings of the National Academy of Sciences of the United States of America, 86: 1831–1835. 2648383 10.1073/pnas.86.6.1831 PMC286798

