# Supplementary material for: Hepatitis delta virus-like circular RNAs from diverse metazoans encode conserved hammerhead ribozymes
Source: Virus Evol. 2021 Feb 18;7(1):veab016. doi: 10.1093/ve/veab016 (PMC7936874; doi:10.1093/ve/veab016)
Supplement: veab016_Supplementary_Data [file veab016_supplementary_data.zip › Supplementary FigS2_leg.pdf]

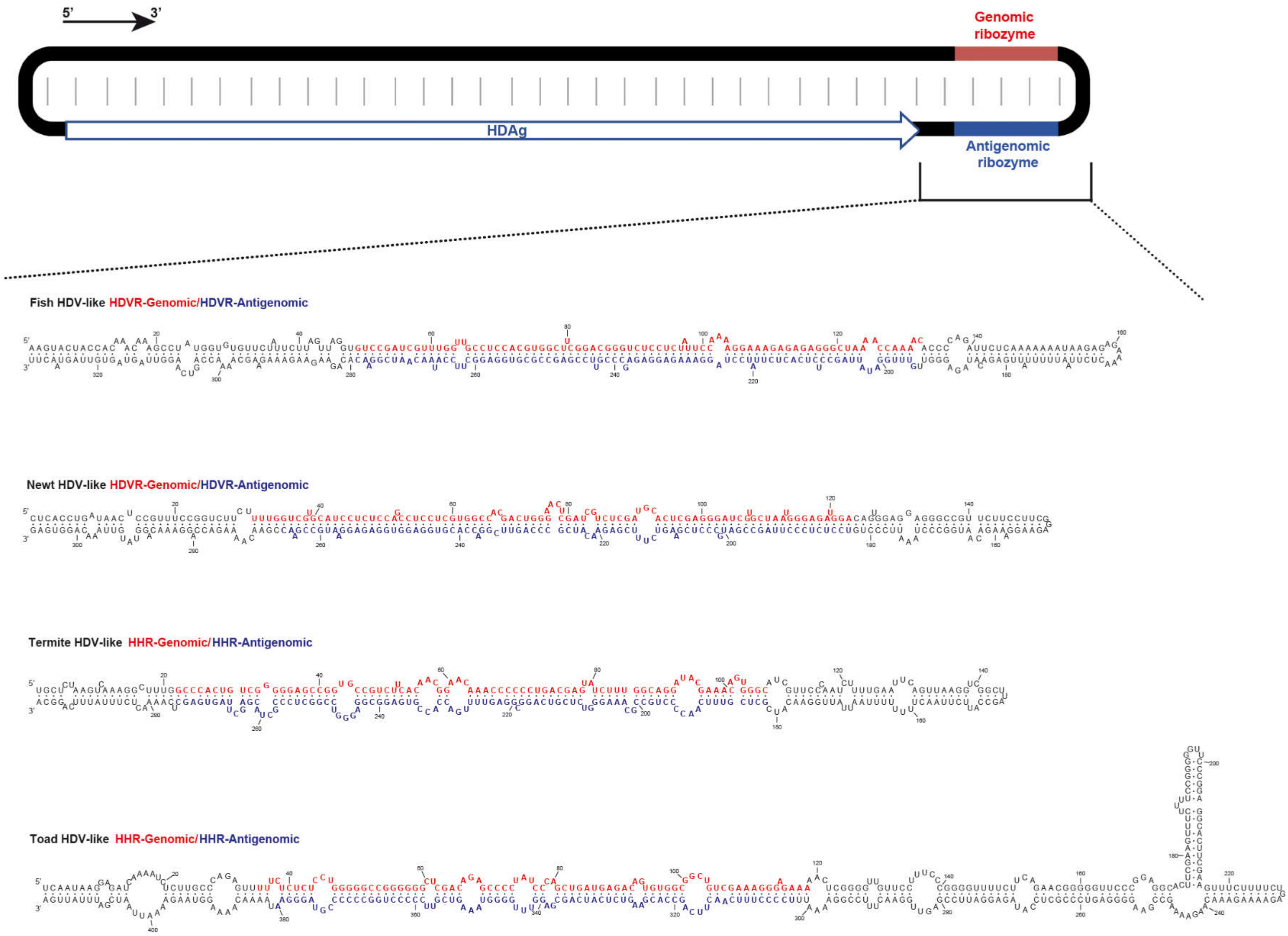

Supplementary Figure S2. Secondary structure predictions of the genomic HDV-like regions (~400 nt) containing the two ribozyme motifs (genomic and antigenomic ribozymes in red and blue, respectively). A schematic representation of the predicted rod-like structures for the human HDV and HDV-like circRNAs is shown at the top.
