# Supplementary material for: Hepatitis delta virus-like circular RNAs from diverse metazoans encode conserved hammerhead ribozymes
Source: Virus Evol. 2021 Feb 18;7(1):veab016. doi: 10.1093/ve/veab016 (PMC7936874; doi:10.1093/ve/veab016)
Supplement: veab016_Supplementary_Data [file Supplementary FigS1_leg.pdf › Supplementary FigS1_leg.pdf]

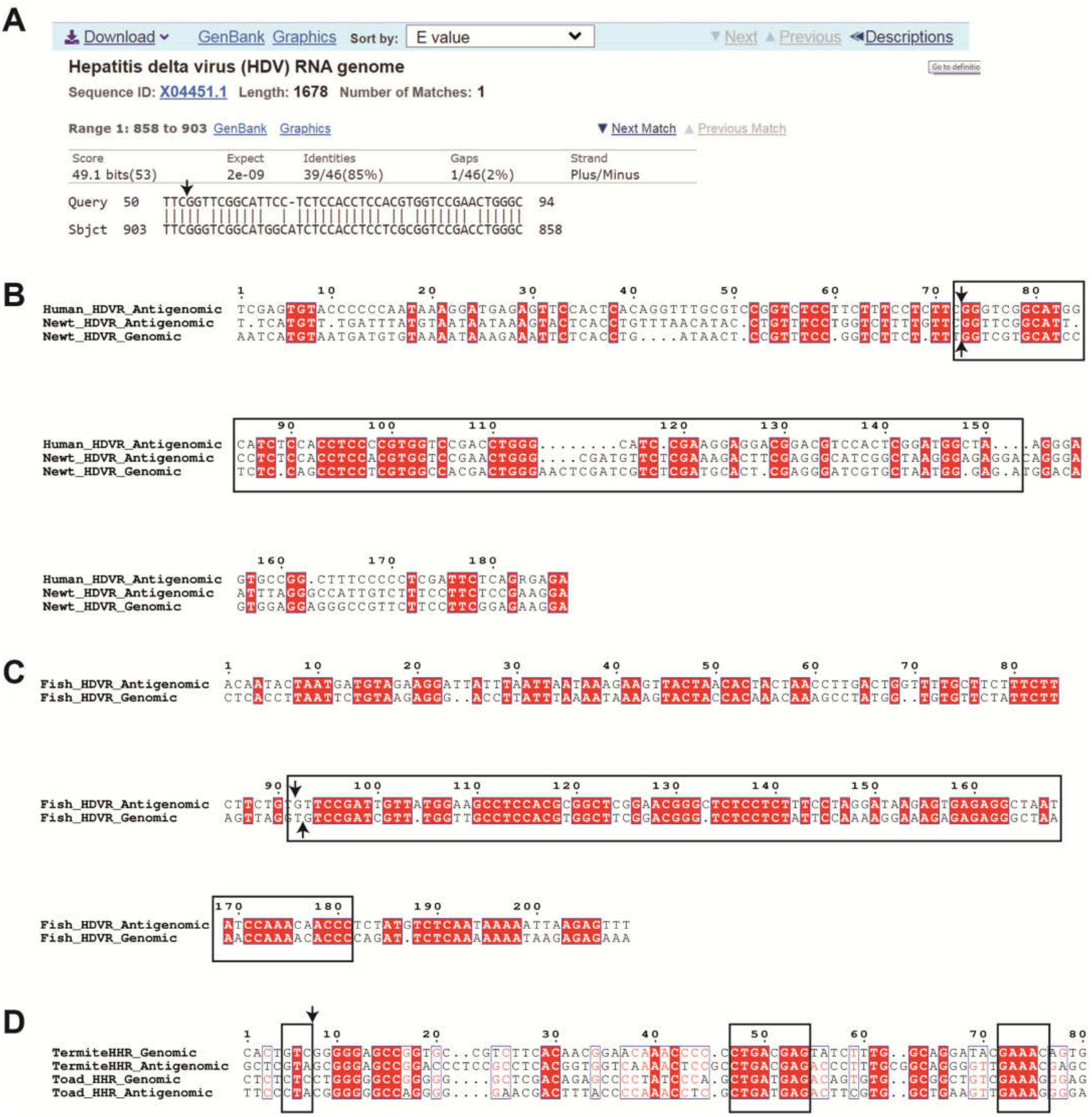

Supplementary Figure S1. (A) BLAST output from a search using as query sequence the 3’ portion (antigenomic) of the termite region indicated in Figure 2B. Sequence alignments of the HDVR of **(B)** newt and **(C)** fish, and **(D)** the HHRs of the toad and termite HDV-like circRNAs. The full HDVRs and the highly conserved HHR sequences are boxed. Sites of self-cleavage are indicated with arrows.
